# GlyGen data model and processing workflow

**DOI:** 10.1093/bioinformatics/btaa238

**Published:** 2020-04-23

**Authors:** Robel Kahsay, Jeet Vora, Rahi Navelkar, Reza Mousavi, Brian C Fochtman, Xavier Holmes, Nagarajan Pattabiraman, Rene Ranzinger, Rupali Mahadik, Tatiana Williamson, Sujeet Kulkarni, Gaurav Agarwal, Maria Martin, Preethi Vasudev, Leyla Garcia, Nathan Edwards, Wenjin Zhang, Darren A Natale, Karen Ross, Kiyoko F Aoki-Kinoshita, Matthew P Campbell, William S York, Raja Mazumder

**Affiliations:** b1 Department of Biochemistry & Molecular Medicine, The George Washington School of Medicine and Health Sciences, Washington, DC 20052, USA; b2 Complex Carbohydrate Research Center, The University of Georgia, Athens, GA 30602, USA; b3 European Bioinformatics Institute, Hinxton CB10 1SD, UK; b4 ZB MED Information Centre for Life Sciences, Cologne 50931, Germany; b5 Department of Biochemistry and Molecular & Cellular Biology, Georgetown University, Washington, DC 20007, USA; b6 Faculty of Science and Engineering, Soka University, Tokyo 192-8577, Japan; b7 Institute for Glycomics Griffith University, Southport QLD 4222, Australia

## Abstract

**Summary:**

Glycoinformatics plays a major role in glycobiology research, and the development of a comprehensive glycoinformatics knowledgebase is critical. This application note describes the GlyGen data model, processing workflow and the data access interfaces featuring programmatic use case example queries based on specific biological questions. The GlyGen project is a data integration, harmonization and dissemination project for carbohydrate and glycoconjugate-related data retrieved from multiple international data sources including UniProtKB, GlyTouCan, UniCarbKB and other key resources.

**Availability and implementation:**

GlyGen web portal is freely available to access at https://glygen.org. The data portal, web services, SPARQL endpoint and GitHub repository are also freely available at https://data.glygen.org, https://api.glygen.org, https://sparql.glygen.org and https://github.com/glygener, respectively. All code is released under license GNU General Public License version 3 (GNU GPLv3) and is available on GitHub https://github.com/glygener. The datasets are made available under Creative Commons Attribution 4.0 International (CC BY 4.0) license.

**Supplementary information:**

Supplementary data are available at *Bioinformatics* online.

## 1 Introduction

This application note introduces the GlyGen data-processing workflow used to build the backend for the GlyGen (York *et al.*, 2019) knowledgebase. This includes detailed information on the molecular, biophysical and functional properties of glycans, genes and proteins organized in pathways and ontologies as well as a rapidly growing body of biological big data related to mutation and expression. All data integrated in the GlyGen project are publicly available in standard formats supported by NCBI (Sayers *et al.*, 2019) and EMBL-EBI (Cook *et al.*, 2018) to promote standardization and sharing of data within the broader glycomics community. GlyGen is a five-star linked open data compliant knowledgebase and a registered member of FAIRsharing.org fulfilling BioDBcore requirements (https://fairsharing.org/biodbcore-001375/).

## 2 Data integration workflow

**Fig. 1.**
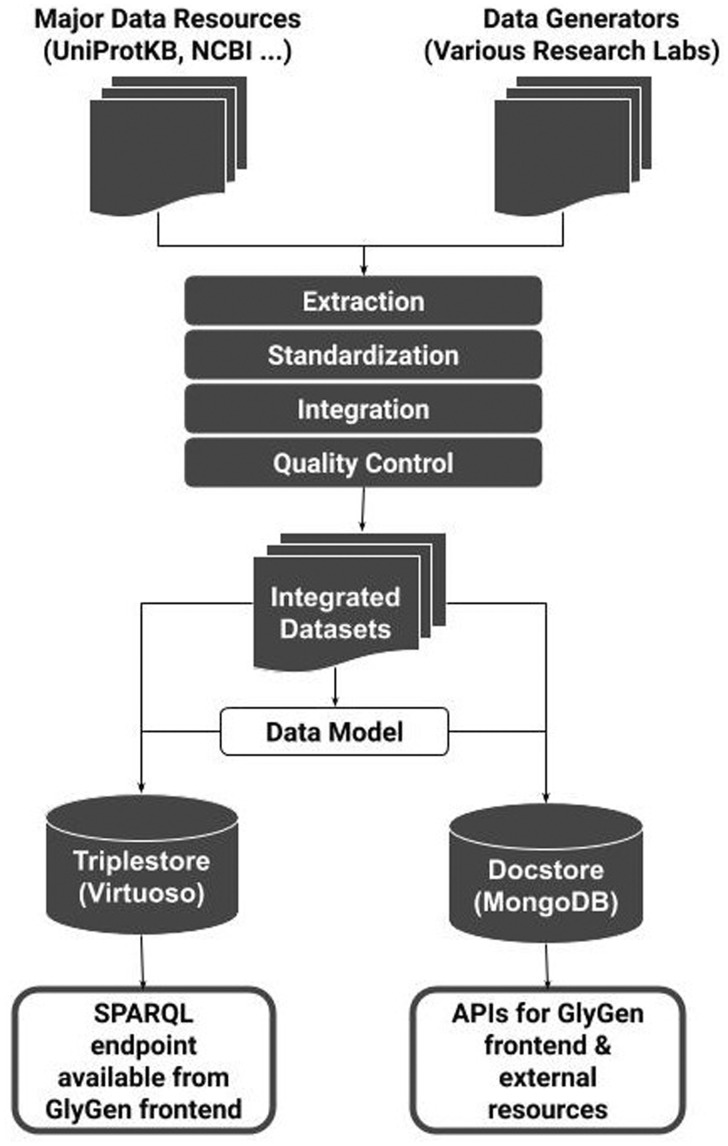
GlyGen data processing workflow showing various steps. Data are retrieved from various resources including UniProtKB, GlyTouCan, UniCarbKB, RefSeq and other key resources, followed by extraction and filtering based on relevance to glycobiology. Extracted data are integrated after harmonization that is based on various standard ontologies. The resulting datasets are then ingested into a MongoDB docstore and Virtuoso triplestore using the GlyGen data model

The framework used to integrate GlyGen data starts by collecting glycan, protein and glycoprotein datasets from major data resources and data generators. The collected heterogeneous datasets are processed following the workflow shown in Figure 1.

### 2.1 Data sources

In GlyGen, GlyTouCan (Tiemeyer *et al.*, 2017) and PubChem (Kim *et al.*, 2016) provide glycan-related data, whereas protein-related data are collected from resources, such as UniProtKB (UniProt Consortium, 2019), NCBI Reference Sequence (RefSeq) (O’Leary *et al.*, 2016), BioMuta (Dingerdissen *et al.*, 2018), BioXpress (Dingerdissen *et al.*, 2018), Mouse Genome Institute (Bult *et al.*, 2019), Orthologous Matrix (Altenhoff *et al.*, 2018), Disease Ontology (Kibbe *et al.*, 2015), Genomics England PanelApp (Martin *et al.*, 2019) and the Monarch Initiative (Mungall *et al.*, 2017). Finally, glycoprotein-related data are integrated from UniCarbKB (Campbell *et al.*, 2014), PDB (Berman *et al.*, 2000) and UniProtKB. In addition to these major resources mentioned, other relevant datasets are collected from various research laboratories.

### 2.2 Dataset standardization, integration and quality control

Data downloaded from various resources are versioned and stored in the GlyGen backend server. The GlyGen knowledgebase maintains strict protein (UniProtKB canonical accessions) and glycan (GlyTouCan accessions) lists, which are used as the protein and glycan primary keys, respectively. Each dataset is mapped to one of these primary keys, and any non-standard identifiers are mapped to their equivalent standard identifiers. Datasets pass through quality control and filtering steps, such as file format sanity, primary accession checks, residue or amino acid position accuracy and various other filtering steps outlined by subject matter experts. The processed dataset is then assigned a GlyGen dataset identifier, and a dataset BioCompute Object (BCO) (Alterovitz *et al.*, 2018) is created to provide detailed documentation of the data-processing workflow. These datasets can be searched, browsed and downloaded from the GlyGen data page (https://data.glygen.org). A detailed description of data preprocessing and normalization for glycan, protein and glycoprotein datasets is given in the Supplementary Texts S1–S3, the GlyGen data page and dataset sample view page are shown in the Supplementary Figure S1a and b.

### 2.3 Biocompute Objects for GlyGen datasets

The dataset BCOs are created in conformance to the current BCO specifications (1.3.0) (https://github.com/biocompute-objects/BCO_Specification/tree/master/schemas). A dataset BCO is created with the data integration process perspective to enable capturing all the metadata related to the processing steps performed in the workflow. The dataset BCOs constructed this way can be used as a ‘readme’ for the dataset that provides precise details on how the dataset is integrated. The use of the BCO standard facilitates granular tracking of metadata especially the provenance that helps in providing appropriate attribution and license information that dictates the usage of the dataset, workflow exchange between the researchers and the reproducibility of the dataset. These dataset BCOs are recorded and represented in machine-readable JavaScript Object Notation (JSON) format and can be viewed and downloaded from the GlyGen data page (https://data.glygen.org).

### 2.4 GlyGen docstore and web services

As mentioned earlier, the GlyGen data integration workflow creates glycan, protein and glycoprotein centric datasets. These datasets are used to generate glycan, protein and glycoprotein centric JSON objects, which are stored in a MongoDB docstore. The GlyGen docstore is used as a backend for various GlyGen web services that are used by the GlyGen frontend as well as other external applications. The GlyGen web services (https://api.glygen.org), which have been documented using the Swagger framework (https://swagger.io/) allow programmatic access of GlyGen data objects for glycans, proteins and glycoproteins. Some of these web services are generic and provide searching, listing and detailed record access functionalities for GlyGen data objects, while others are custom designed to respond to specific biological questions or use cases collected from the user community. The GlyGen API’s webpage is shown in the Supplementary Figure S2.

## 3 GlyGen data model, triplestore and SPARQL endpoint

All data in the GlyGen project are also available in the Resource Description Framework (RDF) format using namespace from various existing ontologies. The UniProt Core Ontology (Redaschi and UniProt Consortium, 2009) and GlyGen Ontology are used to describe protein-centric data whereas glycan-centric data are described using the GlycoRDF Ontology (Ranzinger *et al.*, 2015). The GlyGen Ontology along with the Glycoconjugate Ontology (https://github.com/glycoinfo/GlycoCoO) provides the necessary namespace to represent glycoprotein data.

A partial view of the GlyGen data model is given in the Supplementary Figure S4, showing a glycoprotein entry linked to a protein sequence and one or many glycosylation sites. A glycosylation site consists of an exact or fuzzy position on a protein sequence that is known to have been glycosylated by a glycan or glycan set. An exact glycosylation site position is linked to the amino acid type that occupies it. The GlyGen knowledgebase uses a Virtuoso triplestore to store GlyGen triple data, and a SPARQL Protocol and RDF Query Language (SPARQL) endpoint (https://sparql.glygen.org) is built to provide programmatic access to the triplestore. The webpage for the GlyGen SPARQL interface and triplestore content statistics for release-1.5 are shown in the Supplementary Figure S3 and Table S1, respectively.

## 4 Conclusion

This application note has introduced the data model and processing workflow used for building the backend for the GlyGen knowledgebase. In this processing and integration workflow, data are retrieved and extracted from a number of resources and then standardized and harmonized to create clean high-quality datasets. The dataset creation process is fully documented in a form of metadata by creating BCOs. These datasets are further processed to create JSON objects and RDF triples that populate MongoDB docstore and Virtuoso triplestore backend databases, respectively. The docstore is used by various GlyGen web services while the triplestore is accessed through the GlyGen SPARQL endpoint.

## Supplementary Material

btaa238_Supplementary_Data
