# Supplementary material for: GlyGen data model and processing workflow
Source: Bioinformatics. 2020 Apr 23;36(12):3941–3. doi: 10.1093/bioinformatics/btaa238 (PMC7320628; doi:10.1093/bioinformatics/btaa238)
Supplement: btaa238_Supplementary_Data [file btaa238_supplementary_data.zip › btaa238-Suppl_Data/GlyGenSupplementaryMaterialRevised.docx]

**GlyGen data model and processing workflow**

Robel Kahsay^1,*^, Jeet Vora^1^, Rahi Navelkar^1^, Reza Mousavi^1^, Brian Fochtman^1^, Xavier Holmes^1^, Nagarajan Pattabiraman^1^, Rene Ranzinger^2^, Rupali Mahadik^2^, Tatiana Williamson^2^, Sujeet Kulkarni^2^, Gaurav Agarwal^2^, Maria Martin^3^, Preethi Vasudev^3^, Leyla Jael Garcia Castro^4^, Nathan Edwards^5^, Wenjin Zhang^5^, Darren A. Natale^5^, Karen Ross^5^, Kiyoko F. Aoki-Kinoshita^6^, Matthew P. Campbell^7^, William S. York^2^, Raja Mazumder^1^.

^1^Department of Biochemistry & Molecular Medicine, The George Washington School of Medicine and Health Sciences, Washington, DC, 20052, USA. ^2^Complex Carbohydrate Research Center, The University of Georgia, Athens, GA, 30602, USA. ^3^European Bioinformatics Institute, Hinxton, Cambridgeshire, CB10 1SD, UK. ^4^ZB MED Information Centre for Life Sciences, Cologne, 50931 Germany. ^5^Department of Biochemistry and Molecular & Cellular Biology, Georgetown University, 20007, Washington, DC, USA. ^6^Faculty of Science and Engineering, Soka University, Tokyo, 192-8577, Japan. ^7^Institute for Glycomics, Griffith University, Southport, QLD, 4222, Australia.

*To whom correspondence should be addressed.

Abstract

Summary: Glycoinformatics plays a major role in glycobiology research, and the development of a comprehensive glycoinformatics knowledgebase is critical. This application note describes the GlyGen data model, processing workflow and the data access interfaces featuring programmatic use case example queries based on specific biological questions. The GlyGen project is a data integration, harmonization, and dissemination project for carbohydrate and glycoconjugate related data retrieved from multiple international data sources including UniProtKB, GlyTouCan, UniCarbKB and other key resources.

**Availability and Implementation:** GlyGen web portal is freely available to access at https://glygen.org. The data portal, web services, SPARQL endpoint, and GitHub repository are also freely available at https://data.glygen.org, https://api.glygen.org, https://sparql.glygen.org, and https://github.com/glygener, respectively. All code is released under license GNU General Public License version 3 (GNU GPLv3) and is available on GitHub https://github.com/glygener. The datasets are made available under Creative Commons Attribution 4.0 International (CC BY 4.0) license

**Contact:** rykahsay@gwu.edu

**Supplementary Material**

The Supplementary Material contains the screenshots of the GlyGen’s data access interfaces, a figure showing the partial view of the GlyGen data model, a table showing the GlyGen triplestore’s data content statistics and the detailed description of the data preprocessing and normalization for glycan, protein and glycoprotein datasets in GlyGen

**Table of Contents**

[Figure S1a: GlyGen Data](#_Toc36120706) Interface

[Figure S1b: GlyGen Dataset Sample View](#_Toc36120707)

[Figure S2: GlyGen APIs Webpage](#_Toc36120709)

[Figure S3: GlyGen SPARQL](#_Toc36120710) Interface

[Figure S4: Partial View of the GlyGen Data Model](#_Toc36120712)

[Table S1: Triplestore Data Content Statistics](#_Toc36120713)

[Text S1: Glycans](#_Toc36120714)

[Text S1a: Glycan Sequence](#_Toc36120715)

[Text S1b: Glycan Images](#_Toc36120716)

[Text S1c: Glycan Molecular Weights](#_Toc36120717)

[Text S1d: Glycan Classification](#_Toc36120718)

[Text S1e: Glycan Monosaccharide Composition](#_Toc36120719)

[Text S1f: Glycan Taxonomy ID and Species Annotations](#_Toc36120720)

[Text S1g: Glycan Cross-References and Publications](#_Toc36120721)

[Text S1h: Glycan Motif Associations](#_Toc36120722)

[Text S1i: Putative Glycoenzyme Associations](#_Toc36120723)

[Text S1j: Glycan Subsumption](#_Toc36120724)

[Text S1k: Glycan Structure](#_Toc36120725)

[Text S2: Glycoproteins](#_Toc36120726)

[Text S3: Proteins](#_Toc36120727)

[Text S3a: Protein Data from SIB, UniProt and other EBI resources](#_Toc36120728)

[Text S3b: UniProtKB Annotation](#_Toc36120729)

[Text S3c: Stringent QC Process, Documentation, Releases in Processing Data](#_Toc36120730)

[Text S3d: Format of Data, Availability on FTP Server](#_Toc36120731)

[Text S3e: Inclusion of PMIDs and ECO codes for all Annotations](#_Toc36120732)

[Supplementary References](#_Toc36120733)

## Figure S1a: GlyGen Data Interface


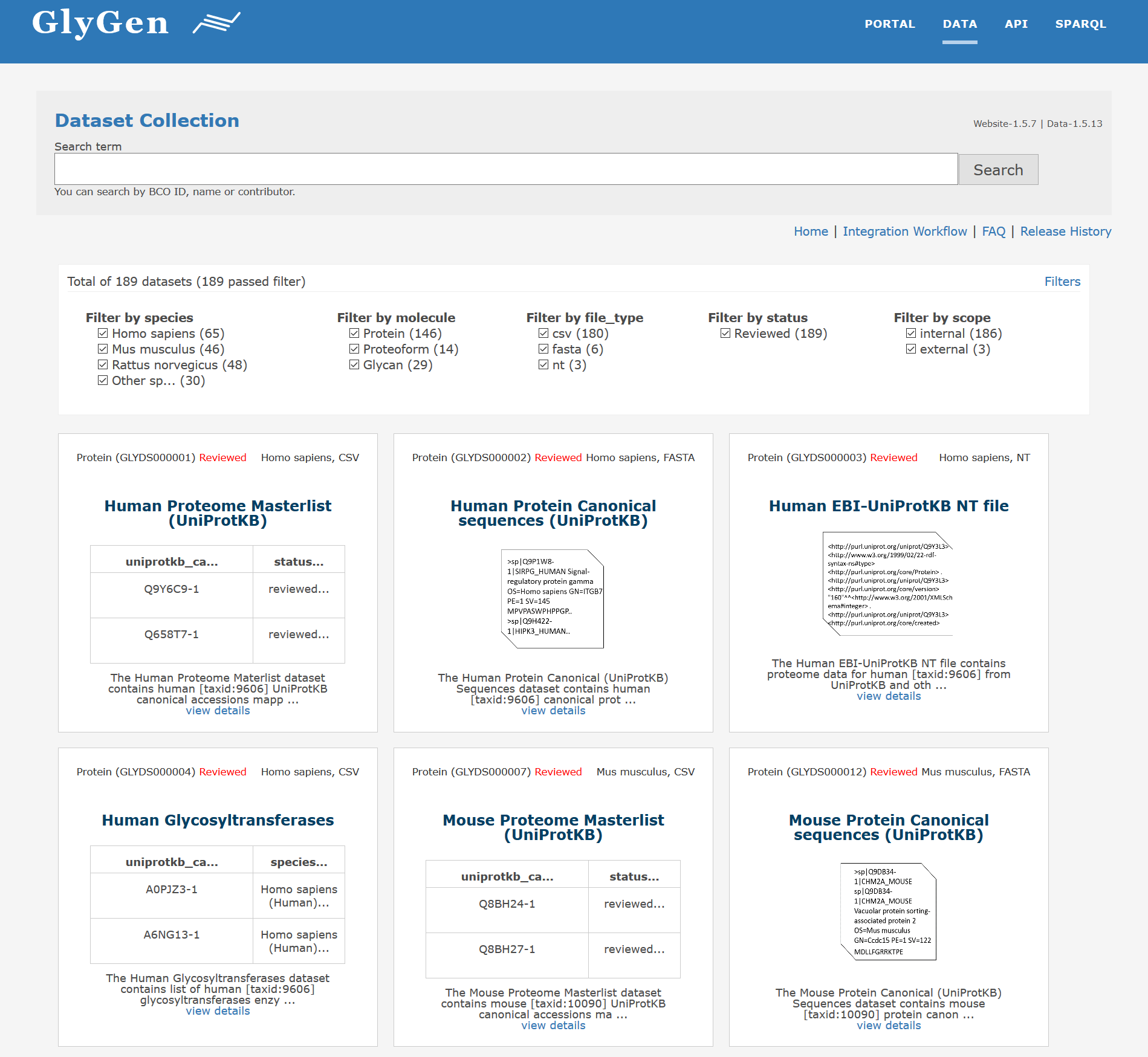


Figure S1a. Screenshot of the GlyGen data interface (<https://data.glygen.org>) (Version – Website-1.5.7 | Data-1.5.13), containing the processed glycan, protein, and glycoprotein (proteoform) datasets. Each dataset is assigned a GlyGen dataset identifier and a dataset BioCompute Object (BCO) is created to provide detailed documentation of the data processing workflow of the dataset. The data page allows searching of datasets by BCO IDs, dataset name and by contributors of the dataset. A filtering option is also provided to filter datasets as per species, macromolecule, filetype, status and by scope. The page also contains links to other pages such as Integration Workflow, FAQ and Release History, providing more information to the users about the data and data processing workflow of GlyGen. Clicking on any dataset opens the sample view page of the dataset.

## Figure S1b: GlyGen Dataset Sample View

##
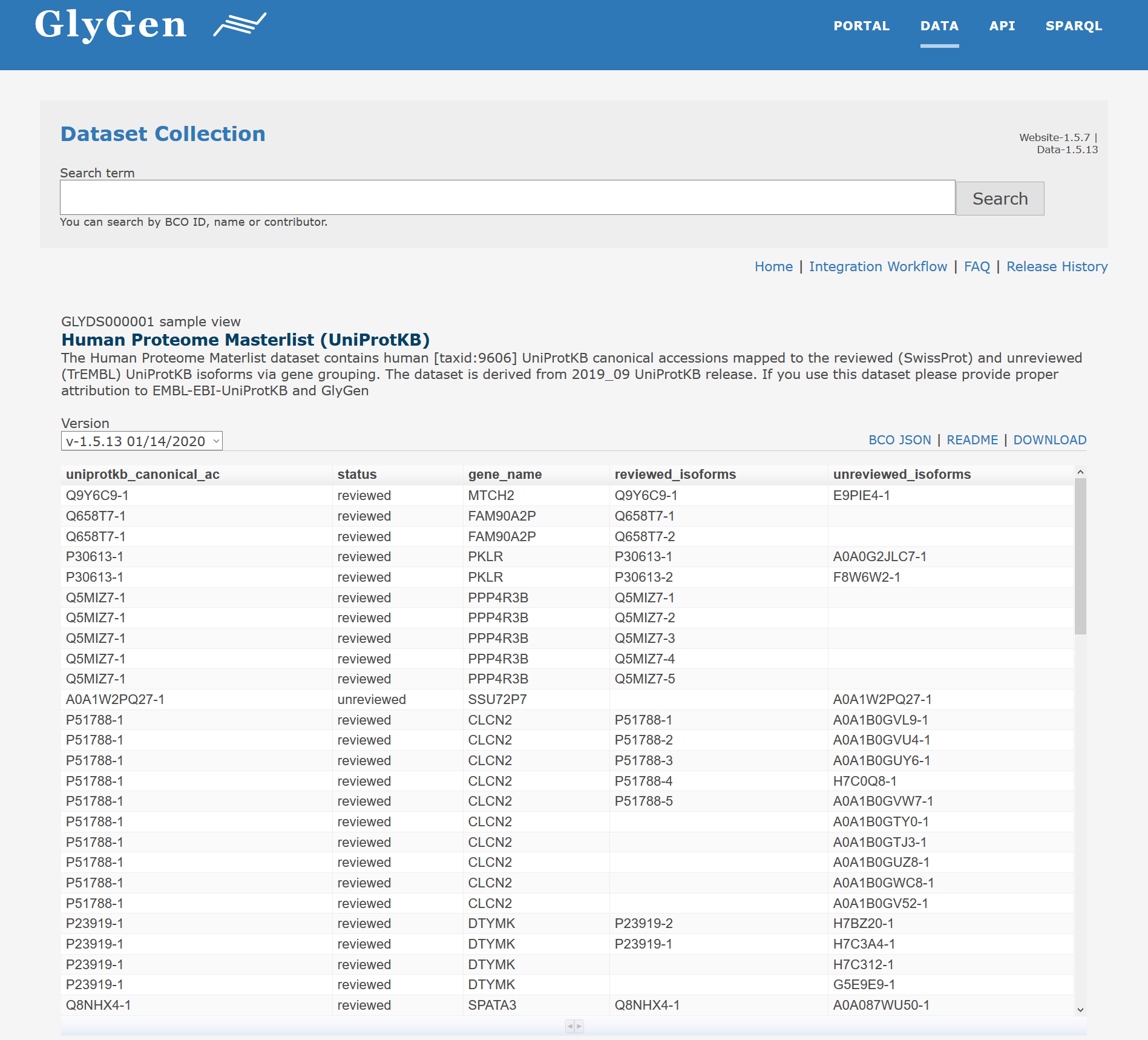


Figure S1b. Screenshot of the GlyGen Human Proteome Masterlist (UniProtKB) dataset sample view page (<https://data.glygen.org/GLYDS000001>) (Version - Website-1.5.7 | Data-1.5.13). The sample view of the dataset contains a short description of the dataset, version details of the dataset and a csv table showing few data entries of the dataset. The sample view page of the dataset also contains the links to the dataset BCO in the JSON format and the README in a human-readable text format that is derived from the JSON dataset BCO. Both the JSON dataset BCO and the README provide detailed data processing workflow of the dataset. The dataset can be downloaded by clicking the DOWNLOAD option.

## Figure S2: GlyGen APIs Webpage


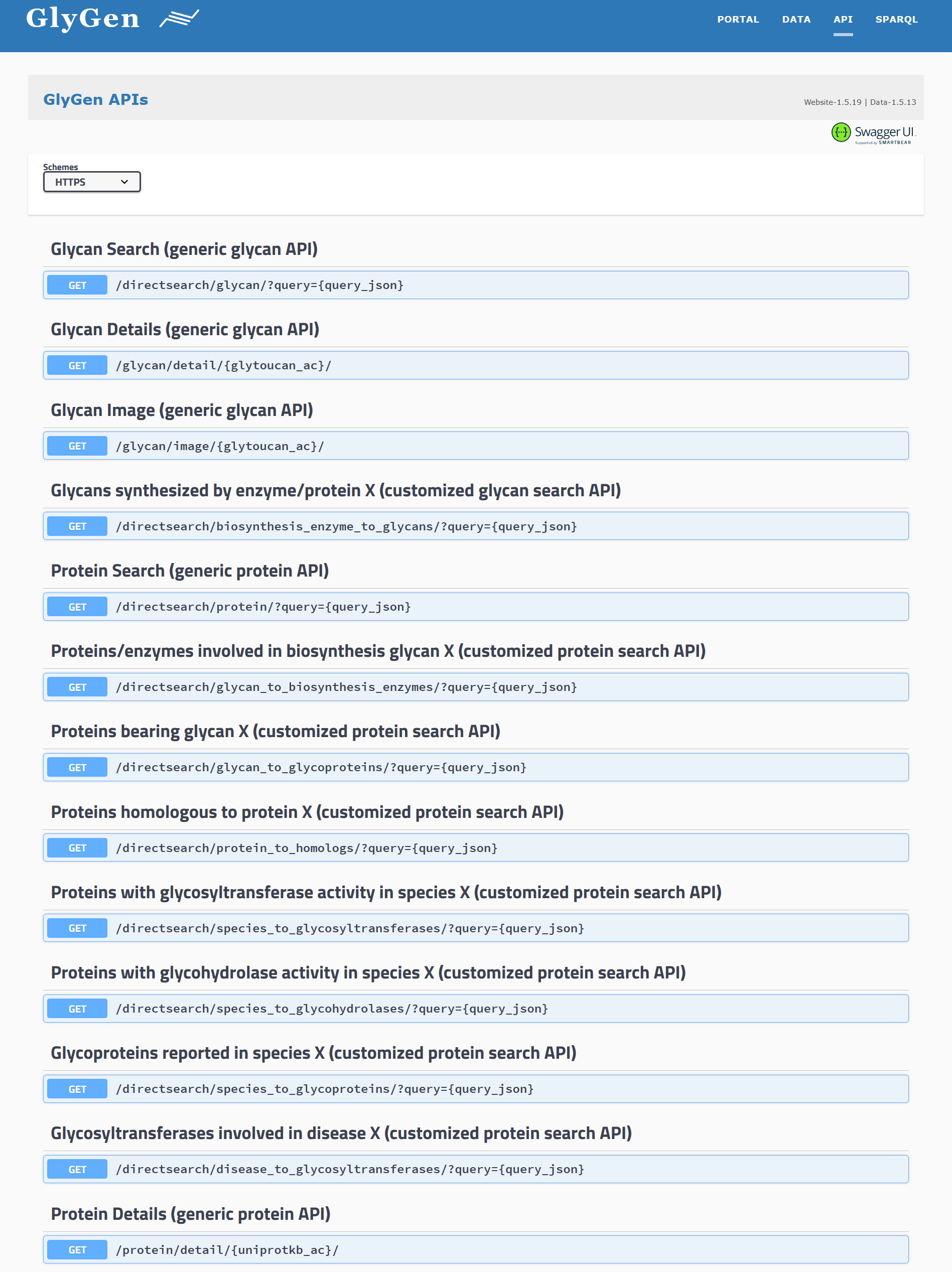


Figure 2. Screenshot of the GlyGen APIs (Application Programming Interface) webpage (<https://api.glygen.org>) (Version - Website-1.5.7 | Data-1.5.13). The GlyGen APIs allow programmatic access of the GlyGen data objects for glycans, proteins, and glycoproteins. The GlyGen APIs have been documented using the Swagger framework (<https://swagger.io/>). Some of these web services are generic and provide searching, listing and detailed record access functionalities for GlyGen data objects, while others are custom designed to respond to specific biological questions or use cases collected from the user community.

## Figure S3: GlyGen SPARQL Interface

##
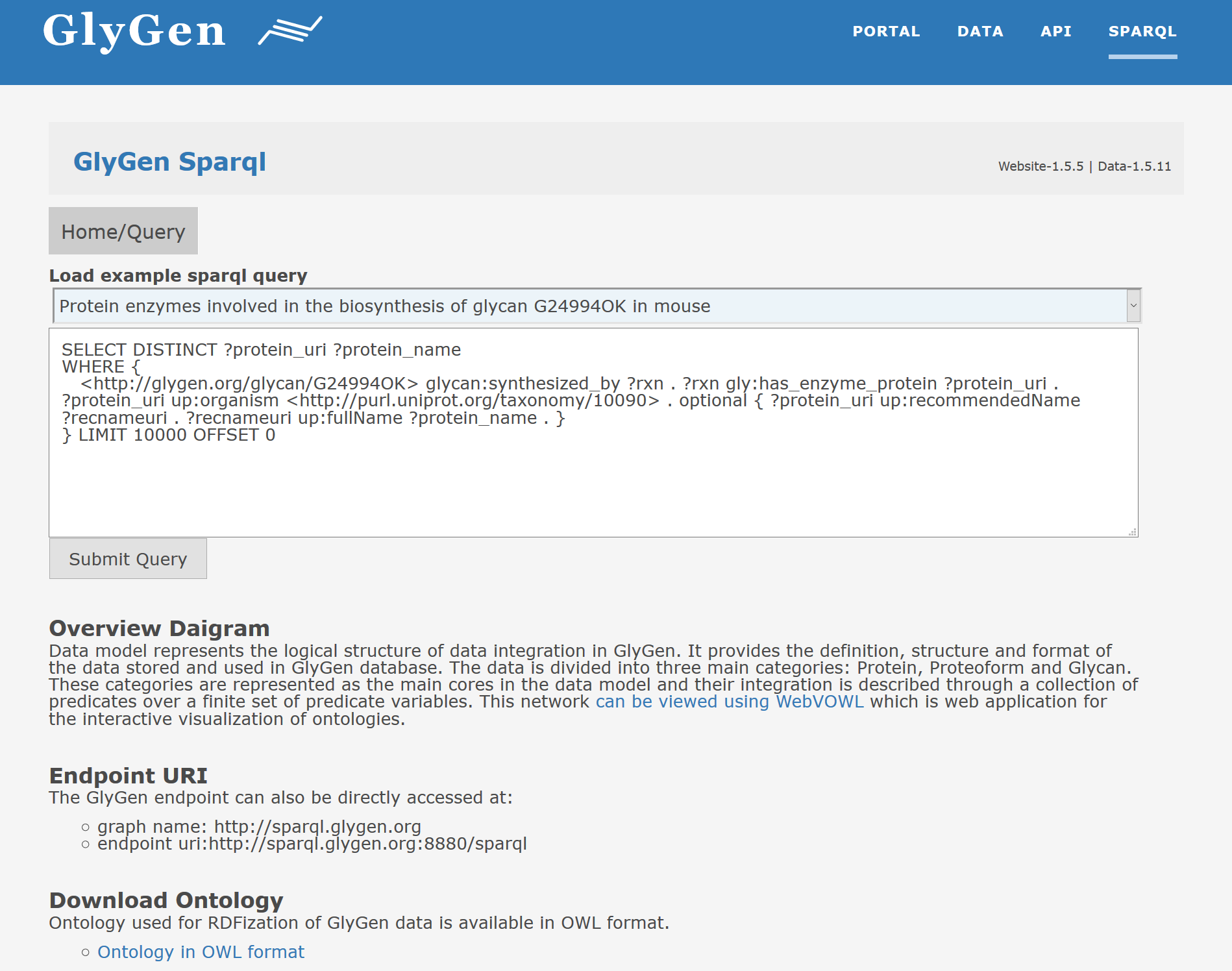


Figure 3. Screenshot of the GlyGen SPARQL (SPARQL Protocol and RDF Query Language ) interface (<https://sparql.glygen.org>) (Version - Website-1.5.7 | Data-1.5.13). The GlyGen SPARQL interface allows programmatic access to the GlyGen data that is available in the Resource Description Framework (RDF) format. The webpage contains sample SPARQL queries allowing users to try and explore the data using the SPARQL query language. The webpage also provides a link to WebVOWL application to view the interactive GlyGen data model developed by using namespace from various existing ontologies. The webpage provides the GlyGen SPARQL endpoint URIs to access the endpoint directly and also provides a link to the download the GlyGen data model ontologies in the OWL format.

## Figure S4: Partial View of the GlyGen Data Model


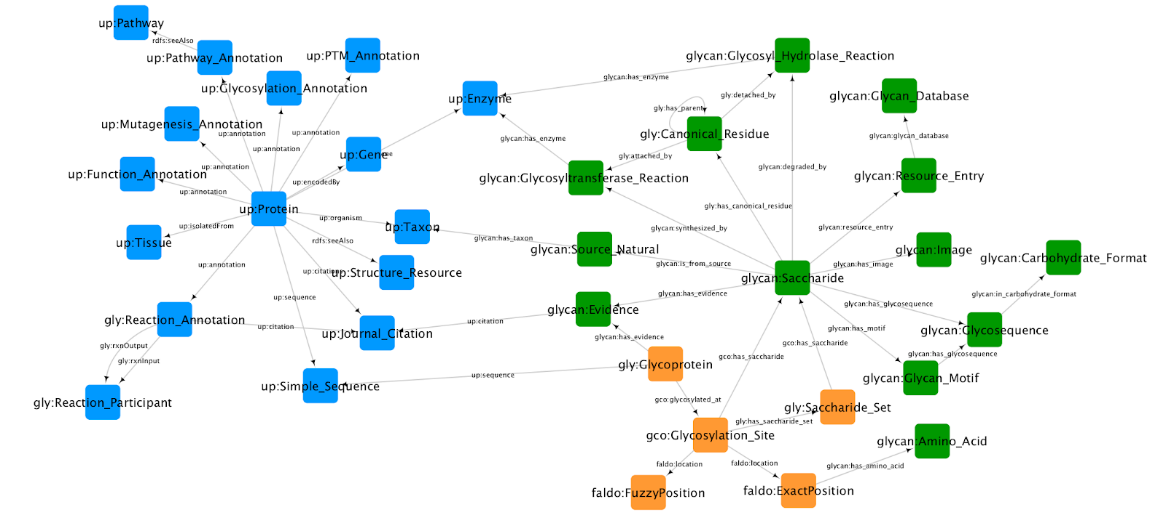


Figure S4. Partial view of the GlyGen data model, the full version can be downloaded in OWL format from https://sparql.glygen.org. The blue nodes in the graph are from the UniProt Core Ontology and GlyGen Ontology while green nodes are the classes from GlycoRDF Ontology. The orange nodes are classes from the GlyGen Ontology and the Glycoconjugate Ontology.

## Table S1: Triplestore Data Content Statistics

The GlyGen triplestore content statistics for release-1.5 are shown in Supplementary Table S1 below, which gives the number of instances corresponding to the major classes or nodes shown in the GlyGen data model.

Table S1: Statistics for instances of major classes

| Class | Instances |
| --- | --- |
| up:Protein | 159128 |
| up:Simple_Seqence | 191004 |
| up:Gene | 155625 |
| up:Enzyme | 1524 |
| up:Glycosylation_Annotation | 38740 |
| glycan:Saccharide | 29290 |
| glycan:Glycosequence | 91082 |
| gly:Glycoprotein | 7341 |
| gco:Glycosylation_Site | 7341 |

## Text S1: Glycans

GlyGen (York, et al., 2019) glycans capture integrated normalized glycan metadata from a variety of different glycoinformatics resources, but especially GlyTouCan (Tiemeyer, et al., 2017) and UniCarbKB (Campbell, et al., 2014)

All glycan entities in GlyGen are referenced by their GlyTouCan accession. The GlyTouCan triplestore (ts.glytoucan.org) provides comprehensive access to its underlying glycan metadata based on the GlyTouCan accession. UniCarbKB data is extracted both from its triplestore (http://sparql.unicarbkb.org) and is supplied directly to the GlyGen project as flat-files.

The cleaned and integrated glycan metadata is publicly available from the Edwards lab glycan data site: http://grg.tn/GlycanData and from the associated triplestore.

### Text S1a: Glycan Sequence

GlyGen glycans are furnished with WURCS 2.0 (Matsubara, et al., 2017), GlycoCT (Herget, et al., 2008), extended IUPAC (McNaught, 1997), and GLYCAM compatible glycan sequences. WURCS 2.0, GlycoCT, and extended IUPAC format sequences are obtained directly from GlyTouCan, where available. The Edwards lab provides GlycoCT format sequences when these are not provided by GlyTouCan and GLYCAM Compatible sequences for fully-defined structures. Importantly, the generation of GlycoCT sequence necessitates the comprehensive ability to parse WURCS 2.0 format sequence, which is consistently available for GlyTouCan glycans.

**Text S1b: Glycan Images**

GlyGen glycans are furnished with static PNG images in extended CFG notation downloaded from the corresponding GlyTouCan web-service, or from the GlyTouCan triplestore.

**Text S1c: Glycan Molecular Weights**

GlyGen glycans have underivatized and permethylated molecular weights computed directly from the glycan sequence by the Edwards lab, as well as underivatized molecular weights accessed from the GlyTouCan triplestore. Anomalies between GlyTouCan and GlyGen underivatized molecular weights suggest semantic differences in WURCS 2.0 and GlycoCT glycan structure representations

**Text S1d: Glycan Classification**

GlyGen glycans are annotated with type and subtype classifications, based primarily on association with specific N-glycan and O-glycan core motifs. However, since these motifs constrain only the core monosaccharides of glycan structures, additional rules and filtering are needed for the correct assignment of semantically accurate glycan subtypes. For N-linked glycans, association with both N-linked hybrid and N-linked high mannose core motifs should be resolved as N-linked hybrid; association with the N-linked high mannose core motif must be filtered by checking the monosaccharide composition to ensure all but two monosaccharides are mannose; while association with O-linked core 1, 2, and 6 core motifs should be resolved as O-linked core 2; and O-linked core 3, 4, and 6 core motifs should be resolved as O-linked core 4. Lastly, specific rules are required to identify paucimannose structures, defined as 3-6 monosaccharides of the N-linked glycan core consisting of two GlcNAc and up to three mannose residues, optionally with a core fucose residue.

**Text S1e: Glycan Monosaccharide Composition**

To support the searching of glycan structure by their monosaccharide composition, we determine the number of monosaccharides that resolve to each IUPAC symbol from the set: Man, Gal, Glc, Xyl, Fuc, ManNAc, GlcNAc, GalNAc, NeuAc, NeuGc, GlcA, GalA, ManA, GlcN, GalN, ManN. The Xxx symbol is used to indicate monosaccharides that do not resolve to an IUPAC symbol. The stereochemistry absent forms Hex, HexNAc, dHex, Pent, Sia, HexA, HexN are also computed as the sum of monosaccharide counts for those with defined stereochemistry and those without. Finally, phosphate, methyl, sulfate, and alditol substituents are counted as distinct entities, matching the convention adopted by others in the glycoinformatics community. These monosaccharide compositions also support rule-based glycan classification and rule-based species determination.

**Text S1f: Glycan Taxonomy ID and Species Annotations**

GlyGen glycans derive their taxonomy id annotations from GlyTouCan and UniCarbKB, whose lists are merged. Additional rules filter out species annotations for structures containing monosaccharide residues that are not available in the species of interest – for example, we require that human glycans have taxonomy id 9606 and no NeuGC or Xyl monosaccharides. Glycans without direct taxonomy ID annotations may still be considered human, mouse, or rat due to subsumption – in this case, a more specific form of the glycan structure description has an explicit species annotation.

**Text S1g: Glycan Cross-References and Publications**

Glycan cross-references to various other glycan resources are captured for GlyGen too. Most of the cross-references come from the GlyTouCan triplestore – these are provided by GlyTouCan partner sites. In addition, some cross-references are provided directly from the referenced resource (UniCarbKB); provided by other glycoinformatics groups (esp. the Aoki-Kinoshita, Campbell, and Ranzinger labs, incl. ChEBI (Hastings, et al., 2016), PubChem (Kim, et al., 2016), and GlycO (Eavenson, et al., 2015) or derived from other information sources. Links to MonosaccharideDB (Lütteke and Lieth, 2005) are computed in the Edwards lab. PubMed IDs (PMIDs) (Sayers, et al., 2019) are extracted from GlyTouCan and UniCarbKB resources and merged.

**Text S1h: Glycan Motif Associations**

Associations between glycan motifs and their structures are extracted from the GlyTouCan triplestore, and as such, the current glycan motif set matches those from the current GlyTouCan set. A comprehensive glycan motif site (http://grg.tn/GlycoMotif) has been prepared to organize and name the different glycan motifs in use in the glycoinformatics community, ultimately, GlyGen will develop its own glycan motif set to support semantic searching and annotation needs.

**Text S1i: Putative Glycoenzyme Associations**

The York lab computes alignments between all fully-determined glycan structures in GlyGen and the canonical monosaccharide residues of the GlycO mammalian N-linked glycan tree; provides human-curated associations between human and mouse glycosyltransferases believed to be responsible for adding each canonical residue. We integrate this information with the GlyGen glycans to provide putative glycoenzyme annotations for GlyGen glycans.

**Text S1j: Glycan Subsumption**

Based on the subsumption annotations of GlyTouCan and the GNOme glycan subsumption ontology project, each GlyGen glycan is classified as a saccharide, topology, composition, or base composition. Furthermore, GNOme subsumption relationships are computed between all pairs of glycans to assist in the exploration of related glycans with more, or less, information about specific linkages, etc.

**Text S1k: Glycan Structure**

Specific glycan structure properties are determined and extracted for use by down-stream queries, including whether or not the glycan structure is fully determined, whether or not the glycan structure includes undetermined linkages, and whether or not the glycan structure has links or not.

## Text S2: Glycoproteins

Curated glycoprotein site-specific annotations are provided by UniCarbKB and mapped to GlyGen’s UniProtKB (UniProt, 2019) canonical sequences. The GlyGen quality control/quality assurance (QC/QA) pipeline validates the labeled glycosylation site positions by checking the amino acid and associated glycan types (see Glycan Classification section). The UniCarbKB curation process checks the presence of N-glycan sequon (Asn–X–Ser/Thr; where X is any amino acid except proline) unless reported to be different by the author, and performs structure consistency checks for mammalian glycans based on known biosynthetic rules. Where necessary, curators infer glycan structural features based on the provided information, graphical depiction, and experimental descriptions.

## Text S3: Proteins

The protein data in GlyGen is integrated from several resources and includes gene-protein information, from UniProtKB and NCBI Reference Sequence (RefSeq) (O'Leary, et al., 2016), sequences from UniProtKB and Ensembl (Cunningham, et al., 2019), disease data from Disease Ontology (DO) (Kibbe, et al., 2015), Monarch Initiative (Mungall, et al., 2017), Mouse Genome Institute (MGI) (Bult, et al., 2019), Genomics England PanelApp (Martin, et al., 2019), mutation and expression data from BioMuta and BioXpress (Dingerdissen, et al., 2018) respectively, glycosylation data from UniProtKB, Protein Data Bank (PDB) (Berman, et al., 2000) and UniCarbKB (Campbell, et al., 2014) and pathways and reactions data from Reactome (Fabregat, et al., 2018) and Rhea (Morgat, et al., 2015). The data from these resources are either directly downloaded or contributed by the resource for integration in GlyGen. All the protein data are mapped to the GlyGen’s UniProtKB canonical accessions list which is also the primary key for the protein datasets.

### Text S3a: Protein Data from SIB, UniProt and other EBI resources

The majority of the protein data in GlyGen is integrated from the Swiss Institute of Bioinformatics (SIB) and European Molecular Biology Laboratory- European Bioinformatics Institute (EMBL-EBI) resources that include UniProtKB, Ensembl, Reactome, and Rhea. Data from these resources are prepared for GlyGen’s consumption by UniProtKB that follows GlyGen’s protein data specifications. The specifications document is based on the UniProt RDF (Redaschi and Consortium, 2009) model, an evolving document following the iteration model and GlyGen Ontology. The attributes in the specifications are identified for protein-related components and then implemented, tested, thoroughly quality assured and packaged as a dataset in RDF N-Triples format for GlyGen. The proteome data in the dataset is derived from individual reference proteomes for the organisms in GlyGen - UP000005640 [*Homo sapiens* (human), UP000000589 [*Mus musculus* (mouse)] and UP000002494 [*Rattus norvegicus* (rat)] that includes reviewed (UniProtKB/Swiss-Prot) and unreviewed (UniProtKB/TrEMBL) accessions. To avoid redundancy of information, UniProtKB describes all the protein products encoded by one gene in a given species in a single entry and for every single entry, canonical sequence accession is chosen based on the criteria mentioned at https://www.uniprot.org/help/canonical_and_isoforms. These canonical accessions that comprise of UniProtKB/Swiss-Prot and UniProtKB/TrEMBL form GlyGen’s protein canonical accessions list and serve as primary key for the protein datasets. The final dataset packaged and released for GlyGen contains core UniProtKB proteome data along with genomic coordinates, peptide information, sequence annotation, pathways and reactions information, protein binary interaction information, publication information and cross-references to other databases and resources.

### Text S3b: UniProtKB Annotation

The dataset contains important annotation data that includes but not limited to functional annotation, molecule processing annotation, sequence annotation, structural annotation, post-translational modifications (PTM) annotation, and position-specific annotation, etc. Some of the annotation examples include (1) glycosylation annotation that provides with the information of glycosylation site and glycosylation type of the protein; (2) PTM annotation that describes PTMs, complementing the information provided at the sequence level; and (3) disease annotation that describes any disease associated with a deficiency of a protein**.**

### Text S3c: Stringent QC Process, Documentation, Releases in Processing Data

QC checks include data format and validation tests, tracking the total number of proteins, sequences, and other attributes, the percentage change between releases and data integrity checks. Other checks ensure that every glycosylation annotation has a range, verify the sum of reviewed and unreviewed sequences is the same as the number of sequences, and that all the proteins have sequences.

### Text S3d: Format of Data, Availability on FTP Server

The UniProtKB datasets containing the protein data as per GlyGen’s protein data specifications are available in RDF N-Triples format. Current release datasets for human, mouse & rat are available at http://ftp.ebi.ac.uk/pub/contrib/glygen/current_release/ and the earlier releases are available at http://ftp.ebi.ac.uk/pub/contrib/glygen/previous_releases/

New datasets for GlyGen are generated after every UniProtKB release.

### Text S3e: Inclusion of PMIDs and ECO codes for all Annotations

As GlyGen is an evidence-based resource, nearly all annotations have corresponding evidence represented by PubMed Identifier (PMID), Evidence and Conclusion Ontology (ECO) code (Chibucos, et al., 2017) and source of data to ensure high data fidelity.

## Supplementary References

Berman, H.M.*, et al.* The Protein Data Bank. *Nucleic Acids Res* 2000;28(1):235-242.

Bult, C.J.*, et al.* Mouse Genome Database (MGD) 2019. *Nucleic Acids Res* 2019;47(D1):D801-D806.

Campbell, M.P.*, et al.* UniCarbKB: building a knowledge platform for glycoproteomics. *Nucleic Acids Res* 2014;42(Database issue):D215-221.

Chibucos, M.C.*, et al.* The Evidence and Conclusion Ontology (ECO): Supporting GO Annotations. *Methods Mol Biol* 2017;1446:245-259.

Cunningham, F.*, et al.* Ensembl 2019. *Nucleic Acids Res* 2019;47(D1):D745-D751.

Dingerdissen, H.M.*, et al.* BioMuta and BioXpress: mutation and expression knowledgebases for cancer biomarker discovery. *Nucleic Acids Res* 2018;46(D1):D1128-D1136.

Eavenson, M.*, et al.* Qrator: a web-based curation tool for glycan structures. *Glycobiology* 2015;25(1):66-73.

Fabregat, A.*, et al.* The Reactome Pathway Knowledgebase. *Nucleic Acids Res* 2018;46(D1):D649-D655.

Hastings, J.*, et al.* ChEBI in 2016: Improved services and an expanding collection of metabolites. *Nucleic Acids Res* 2016;44(D1):D1214-1219.

Herget, S.*, et al.* GlycoCT-a unifying sequence format for carbohydrates. *Carbohydr Res* 2008;343(12):2162-2171.

Kibbe, W.A.*, et al.* Disease Ontology 2015 update: an expanded and updated database of human diseases for linking biomedical knowledge through disease data. *Nucleic Acids Res* 2015;43(Database issue):D1071-1078.

Kim, S.*, et al.* PubChem Substance and Compound databases. *Nucleic Acids Res* 2016;44(D1):D1202-1213.

Lütteke, T. and Lieth, C.W. MonoSaccharideDB: A reference resource to unify the notation of carbohydrate residues. 2005.

Martin, A.R.*, et al.* PanelApp crowdsources expert knowledge to establish consensus diagnostic gene panels. *Nat Genet* 2019;51(11):1560-1565.

Matsubara, M.*, et al.* WURCS 2.0 Update To Encapsulate Ambiguous Carbohydrate Structures. *J Chem Inf Model* 2017;57(4):632-637.

McNaught, A.D. International Union of Pure and Applied Chemistry and International Union of Biochemistry and Molecular Biology. Joint Commission on Biochemical Nomenclature. Nomenclature of carbohydrates. *Carbohydr Res* 1997;297(1):1-92.

Morgat, A.*, et al.* Updates in Rhea--a manually curated resource of biochemical reactions. *Nucleic Acids Res* 2015;43(Database issue):D459-464.

Mungall, C.J.*, et al.* The Monarch Initiative: an integrative data and analytic platform connecting phenotypes to genotypes across species. *Nucleic Acids Res* 2017;45(D1):D712-D722.

O'Leary, N.A.*, et al.* Reference sequence (RefSeq) database at NCBI: current status, taxonomic expansion, and functional annotation. *Nucleic Acids Res* 2016;44(D1):D733-745.

Redaschi, N. and Consortium, U. UniProt in RDF: Tackling Data Integration and Distributed Annotation with the Semantic Web. *Nature Precedings* 2009.

Sayers, E.W.*, et al.* Database resources of the National Center for Biotechnology Information. *Nucleic Acids Res* 2019;47(D1):D23-D28.

Tiemeyer, M.*, et al.* GlyTouCan: an accessible glycan structure repository. *Glycobiology* 2017;27(10):915-919.

UniProt, C. UniProt: a worldwide hub of protein knowledge. *Nucleic Acids Res* 2019;47(D1):D506-D515.

York, W.S.*, et al.* GlyGen: Computational and Informatics Resources for Glycoscience. *Glycobiology* 2019.
